# Physical Education of Children

**Published:** 1880-03

**Authors:** 


### PHYSICAL EDUCATION OF CHILDREN.

To this day, the tent is the only dwelling of many of the pastoral tribes of the East. Nothing more is necessary. It is considered more healthful to dwell in tents than in houses. Many families that can afford to do so, are in the habit of forsaking their houses and of dwelling in tents in the fields during the summer months. This is especially true of the inhabitants of their large cities. On account of the narrowness of the streets, and of the filth ahd accumulated rubbish, and perhaps from other causes also, Jerusalem is now considered so sickly, that most Europeans residing there and the more wealthy Turkisk families, are in the habit of passing the summer or early autumn in tents on the surrounding hills.

The late Pasha of Egypt had a palace built in the desert, as one goes from Cairo to Suez, where his stores and harem were lodged, and around which he and his court encamped once a year for several months. A late King of Persia, also was in the habit of leaving his capital every year with his nobles, and more than half of its inhabitants, to encamp in the open air on the plain of Sultanieh. The *exodus* of our cities in the summer time is borrowed, therefore, from the East. And a great pity it is that the model is not more faithfully followed. If, instead of congregating at fashionable watering-places, and crowding badly-ventilated saloons, and sleeping in cells, and keeping late hours, and eating and dressing as if under orders to commit suicide in the most approved manner, the inhabitants of our cities were found upon the mountains and the plains, and compelled to live on plain fare, and sleep in the open air, they would be great gainers in health and beauty, and length of life

As a people the Americans are the most careless, headstrong, and indifferent people about life and health upon earth. They have few manly sports for their boys at school. The exercises of the youth of the English nobility, at Eton, at playing ball and swimming, and the like, which are as much a part of their daily routine as their recitations, are considered amongst us as too *boyish*, even for boys. Our daughters, too, grow up without being able to walk, and many of them without knowing how to ride a horse. Their getting about in the city has to be by *transportation* on wheels. Our children sit and eat and sleep and study too generally in apartments that seem to have been constructed studiously to prevent the admission of pure air. Our assembly-rooms, school-houses and churches are generally built without any reference to a free circulation of fresh air. It is my solemn conviction, from long observation, that many children are made dwarfs, or live pale, emaciated, nervous, consumptive specimens of humanity, and then die before their time, from the want of pure air, more than from any other cause.

Why is it that dyspepsia is an American disease? Why is an American, and especially an American student or clergyman, known at sight in Europe by his pale lean face, and drooping emaciated form? The main reason is, in his youth and in acquiring an education, he has not taken exercise in the open air, and too often his food has been too poor. Why are not the Arabs and the Indians, and the dwellers in tents, the victims of paralysis, gout, rheumatism, dyspepsia, and consumption? The main reason is they live in the open air, and their limbs are strengthened by exercise.

Let our children then starve for bread rather than for air. Let us see to it that their apartments at home and in the school-room are well ventilated, and that they are not too long confined on hard benches in crowded rooms. Let them learn to play as well as to study. Let us educate their bodies as well as their minds. I am always sad when I hear it said that such a boy or girl is a remarkably good child. For by this is generally meant that they are very clever at a book, and that they do not romp and play, and occasionally get a dirty nose or present a torn garment. I am sad, because as a general thing, these *good children* are precocious, and either die before maturity, or drag out a life of feebleness. It is the noise-making child—the stirring child, that developes his physical parts with his mind, that is able at last to make a noise in the world to some purpose. It is very certain we need to pay more attention to physical education. The result is inevitable. If we do not we must degenerate. Our children must have plenty of pure air and of cheerful exercise.

